# First in class dual MDM2/MDMX inhibitor ALRN-6924 enhances antitumor efficacy of chemotherapy in *TP53* wild-type hormone receptor-positive breast cancer models

**DOI:** 10.1186/s13058-021-01406-x

**Published:** 2021-03-04

**Authors:** Seyed Pairawan, Ming Zhao, Erkan Yuca, Allen Annis, Kurt Evans, David Sutton, Luis Carvajal, Jian-Guo Ren, Solimar Santiago, Vincent Guerlavais, Argun Akcakanat, Coya Tapia, Fei Yang, Priya Subash Chandra Bose, Xiaofeng Zheng, Ecaterina Ileana Dumbrava, Manuel Aivado, Funda Meric-Bernstam

**Affiliations:** 1 Department of Surgical Oncology, The University of Texas MD Anderson Cancer Center, Houston, TX USA; 2 Department of Investigational Cancer Therapeutics, The University of Texas MD Anderson Cancer Center, 1400 Holcombe Blvd, Houston, TX 77030 USA; 3 Aileron Therapeutics, Cambridge, MA USA; 4 Department of Translational Molecular Pathology, The University of Texas MD Anderson Cancer Center, Houston, TX USA; 5 Present address: Epizyme Inc., Cambridge, MA USA; 6 Department of Bioinformatics and Computational Biology, The University of Texas MD Anderson Cancer Center, Houston, TX USA; 7 Sheikh Khalifa Bin Zayed Al Nahyan Institute for Personalized Cancer Therapy, The University of Texas MD Anderson Cancer Center, Houston, TX USA; 8 Department of Breast Surgical Oncology, The University of Texas MD Anderson Cancer Center, Houston, TX USA

**Keywords:** MDM2 inhibitor, MDM4 inhibitor, Chemotherapy, Breast cancer, p53

## Abstract

**Background:**

MDM2/MDMX proteins are frequently elevated in hormone receptor-positive (ER+) breast cancer. We sought to determine the antitumor efficacy of the combination of ALRN-6924, a dual inhibitor of MDM2/MDMX, with chemotherapy in ER+ breast cancer models.

**Methods:**

Three hundred two cell lines representing multiple tumor types were screened to confirm the role of *TP53* status in ALRN-6924 efficacy. ER+ breast cancer cell lines (MCF-7 and ZR-75-1) were used to investigate the antitumor efficacy of ALRN-6924 combination. In vitro cell proliferation, cell cycle, and apoptosis assays were performed. Xenograft tumor volumes were measured, and reverse-phase protein array (RPPA), immunohistochemistry (IHC), and TUNEL assay of tumor tissues were performed to evaluate the in vivo pharmacodynamic effects of ALRN-6924 with paclitaxel.

**Results:**

ALRN-6924 was active in wild-type *TP53* (WT-*TP53*) cancer cell lines, but not mutant *TP53*. On ER+ breast cancer cell lines, it was synergistic in vitro and had enhanced in vivo antitumor activity with both paclitaxel and eribulin. Flow cytometry revealed signs of mitotic crisis in all treatment groups; however, S phase was only decreased in MCF-7 single agent and combinatorial ALRN-6924 arms. RPPA and IHC demonstrated an increase in p21 expression in both combinatorial and single agent ALRN-6924 in vivo treatment groups. Apoptotic assays revealed a significantly enhanced in vivo apoptotic rate in ALRN-6924 combined with paclitaxel treatment arm compared to either single agent*.*

**Conclusion:**

The significant synergy observed with ALRN-6924 in combination with chemotherapeutic agents supports further evaluation in patients with hormone receptor-positive breast cancer.

**Supplementary Information:**

The online version contains supplementary material available at 10.1186/s13058-021-01406-x.

## Introduction

Key functions of the cellular tumor suppressor p53 are to prevent the development of malignancy and stop the growth and expansion of abnormal cells through repair of DNA damage, arresting cell cycle, induction of apoptosis, and controlling angiogenesis [1]. During physiological conditions, p53 levels remain low but once activated it is followed by an increase in protein levels, increase in DNA binding, increase in multiple target genes, and activation of its corresponding biological effects [1]. Based on Pan-cancer data, *TP53* remains the most frequently mutated gene observed in human malignancy [2]. p53 is nullified either by alterations in the *TP53* gene or through oncogenic activity of the E3 ubiquitin-protein ligase MDM2 [3] and the related MDMX (MDM4) proteins [4]. Two different models of biological functions of mouse double minute (MDM) proteins have previously been proposed where in the first model, Mdm2 and Mdm4 act independently by regulating specific activities of p53. In this model, it is proposed that p53 levels are mainly being regulated by MDM2, and the transcriptional activity by MDMX. In the second model, they jointly regulate p53 function [4–7].

Amplification of the *MDM2* gene and/or overexpression of its protein have been found to be a main driver of malignancy in many tumor types, especially in tumors with retained wild-type *TP53* status (WT *TP53*) [8]. A previous study demonstrated estrogen receptor-positive (ER+) breast cancer comprises over 70% of breast cancers [9] and majority of ER+ breast cancers have WT *TP53* status [10]. Additionally, 50% of human tumors retain their WT *TP53* status but this differs across tumor histologies [5]. Although not being the key regulator of p53, Mdm4 overexpression and amplification at the gene level has been linked with multiple human malignancies, promoting neoplastic proliferation. This was mainly observed in ER+ breast cancers [11]. MDM2 inhibitors, also known as nutlins, were initially identified in the early 2000s [12] and were found to interrupt the MDM2-p53 interaction. Since then, the portfolio of small molecule MDM2 inhibitors has continued to grow [13]. They have demonstrated promising antitumor efficacy in preclinical ER+ breast cancer [14]. Interestingly, it was found that in order to achieve full p53 activation, dual inhibition of Mdm2 and MDMX is needed [15]. ALRN-6924 is a first of its class cell-penetrating stapled α-helical peptide that disrupts the interaction of p53 tumor suppressor protein and its endogenous inhibitors Mdm4 and Mdm2 in WT *TP53* tumors [16–18]. ALRN-6924 was tolerated and has shown antitumor activity in a phase I clinical trial in patients with solid tumors (NCT02264613) [17].

Paclitaxel is one of the most commonly used standard of care chemotherapeutic agents in breast cancer patients [19]. It induces microtubular polymerization, which leads to stabilization of microtubules, followed by catastrophic mitotic arrest, and demise of the cancer cell [20]. Eribulin is another safe and tolerable FDA-approved anti-microtubule inhibitor which showed an improvement in overall and progression-free survival in metastatic breast cancer patients progressing on prior chemotherapy [21].

Resistance to chemotherapeutic agents is a major concern when treating breast cancer patients, especially with taxanes [22]. MDM2 is a known mediator of chemoresistance [23]; this further accentuates the pressing need to develop rational combinatorial regimens to overcome this resistance. To date, chemotherapeutic agents have not been clinically evaluated in combination with an MDM2/MDMX inhibitor. The aim of the reported studies was to determine if these drug combinations will work synergistically and increase the sensitivity of cancer cells to chemotherapeutic agents. We therefore sought to evaluate the combined antitumor efficacy of ALRN-6924 and paclitaxel or eribulin in ER+ breast cancer with WT *TP53* status by testing cell lines in vitro and in vivo. Our findings indicate that ALRN-6924 acts synergistically with both paclitaxel and eribulin in preclinical breast cancer models.

## Materials and methods

### Reagents

ALRN-6924 was kindly provided by Aileron Therapeutics Inc. (Watertown, MA, USA). RG7112 and RG7388 (idasanutlin) and paclitaxel were purchased for in vitro studies from MedChemExpress (Monmouth Junction, NJ, USA). For in vivo paclitaxel efficacy studies, the agent was obtained from MD Anderson Cancer Center pharmacy. This source of paclitaxel was later changed to the National Cancer Institute for pharmacodynamic experiments. Paclitaxel was prepared in 15% ethanol, 15% kolliphor (Sigma-Aldrich Co., LLC, St Louis, MO, USA), and water for in vivo studies. Eribulin was obtained from the MD Anderson Cancer Center pharmacy. Dimethyl sulfoxide (DMSO) was obtained from Sigma-Aldrich (Sigma-Aldrich Co., LLC, St Louis, MO, USA). All drugs used for in vitro studies were initially prepared in DMSO prior to dilution in a tissue culture medium.

### Cell line panel screening

Two commercial cancer cell line panels, the Eurofins OncoPanel (St. Charles, MO; *n* = 233 cell lines) and a focused Horizons Discovery OncoSignature panel (Cambridge, UK; *n* = 89), were employed. Total 302 tested cell lines representing multiple solid and hematologic tumor types were composed of 100 cell lines with WT *TP53* and 202 cell lines with mutant or null *TP53* (Supplementary Table 1). Cell lines from solid and hematologic tumors were seeded into 384-well plates and incubated in a humidified atmosphere of 5% CO_2_ at 37 °C overnight. Cells were treated with test compounds over 10 concentrations, up to 30 μM, for 72 h. Cells were then fixed and stained in the same wells with nuclear dye and fluorescently labeled antibodies (Eurofins) or ATP levels were measured by adding Perkin Elmer ATPLite (Horizons). Relative cell proliferation was assessed by changes in nuclear dye uptake or ATP levels compared to the untreated cell plate and presented as the effective concentration to inhibit 50% of cell proliferation (IC_50_).

In an independent screen of ER+ breast cancer cell lines, 6 cell lines were used, including MCF-7, ZR-75-1 (WT TP53), and HCC-1954, SK-BR-3, BT-474, CAMA1 (mutant *TP53*). These ER+ breast cancer cell lines were obtained from American Tissue Culture Collection (Manassas, VA, USA). Except for ZR-75-1 cells that were cultured in RPMI-1640 medium, other cell lines were cultured in Dulbecco’s modified Eagle’s medium/F12 supplemented with 10% fetal bovine serum (FBS) at 37 °C and humidified in 5% CO_2_. Cells were seeded in 96-well plates overnight at a density of 5000–10,000 cells per well based on cell line growth characteristics. Cells were treated with ALRN-6924 single agent in a dose range of 0.64–50,000 nM for 3 days. Sulforhodamine B staining (SRB) assay was performed to determine cell survival rate [24]. IC_50_ was determined based on the sigmoid drug-inhibition curve using GraphPad Prism v7.03 software.

### Drug combination assay

Two ER+ breast cancer cell lines with WT *TP53*, MCF-7 and ZR-75-1, were used to determine ALRN-6924 combination efficacy. Cells were treated with ALRN-6924 in combination with chemotherapy agents at individual dose ranges, based on the dose-response curves for ALRN-6924 single drug treatment as described above and for the chemotherapy drugs that we previously established. Each combination treatment group has 6 doses with a fixed combination ratio and a fixed serial dilution. For example, in the combination of ALRN-6924 + paclitaxel, we chose 5000 nM and 10 nM as the highest doses for ALRN-6924 and paclitaxel respectively (combination ratio, 500). Then we had 5 serial dilutions at 5-fold from the highest combo doses. In the combination of ALRN-6924 with eribulin, 5000 nM and 80 nM were chosen as the highest doses respectively (combination ratio, 62.5). To evaluate combination efficacy, a combination index (CI) was determined using the Chou-Talalay drug combination model (CI < 1.0 (curve left-shift): synergistic; CI = 1.0, additive; CI > 1.0 (curve right-shift): antagonistic). Experiments were performed in triplicate and repeated three times.

### Colony formation assay

Cells were seeded in 60-mm plates at a density of 1 × 10^3^ for MCF-7 and 5 × 10^3^ for ZR-75-1 per well. Drug treatment started following 24 h of incubation, with changing of culture media and drugs at least twice a week. Each treatment group was tested in duplicate. Culturing and treatment of cells continued for 2–3 weeks. At the end of the experiment, the colonies were fixed in 10% buffered formalin and stained with 0.05% crystal violet in 25% methanol. Total colony area was measured using NIH ImageJ v1.52a software (NIH, Bethesda, MD, USA).

### Western blot assay

Pierce BCA protein assay kit (ThermoFisher, Waltham, MA, USA) was utilized to obtain protein concentration of cell lysates. Membranes were blocked with 0.1% casein blocking buffer following separation using sodium dodecyl sulfate-polyacrylamide gel electrophoresis and transfer of proteins onto a 0.2-μm nitrocellulose membrane (Bio-Rad Laboratories, Hercules, CA, USA). Next, immunoblotting was performed by probing membranes with antibodies (anti-Mdm2 (#86934), anti-p53 (#9282), anti-p21 (#2947), anti-p-Histone-H3 (Ser10) (#29237), anti-c-Myc (#9402), anti-LC3A/B (#12741), anti-p-Rb (Ser807/811) (#8516), anti-Rb (#9309), anti-p-S6 (Ser235/236) (#4858), and anti-S6 (#2217)). All described antibodies were purchased from Cell Signaling Technology (Boston, MA, USA). Anti-β-actin antibody (#A5441) was purchased from Sigma-Aldrich (Sigma-Aldrich Co., LLC, St Louis, MO, USA). Immunoblotting signals were visualized by Odyssey IR imaging system (Li-Cor Biosciences, Lincoln, NE, USA). Image Studio software v4.0 (Li-Cor Biosciences, Lincoln, NE, USA) was used for the analysis of the bands.

### Annexin V assay

To evaluate for apoptosis, we utilized the Annexin V Apoptosis kit (#11858777001 Roche, Indianapolis, IN, USA). Cells were treated for 72 h with either vehicle, ALRN-6924 10 μM, paclitaxel 1 μM, or combination of both followed by collection of both floating and attached cells. These concentrations were chosen because of findings of p53 reactivation and apoptosis on immunoblotting, respectively. Cells were then probed with both Annexin V and propidium iodide (PI). Labeled cells were analyzed by flow cytometry in the MDACC Flow Cytometry and Cellular Imaging Core. Annexin V-positive cells were counted.

### Cell cycle analysis

To determine the effects of the combination of ALRN-6924 and paclitaxel on the cell cycle, we used MCF-7 and ZR-75-1 cell lines. Cells were plated and treated the following day in duplicates for 24 h with either vehicle, ALRN-6924 10 μM, paclitaxel 1 μM, or combination of both. Samples were collected, fixed, stained with PI (Abcam, #ab139418, Cambridge, MA, USA) according to manufacturer protocol, and analyzed by flow cytometry in the MDACC Flow Cytometry and Cellular Imaging Core Facility. We evaluated the percentages of cells in each cell cycle phase: subG1, G0-G1, S, and G2-M.

### In vivo studies

All animal experiments were approved by the Animal Care and Use Committee of MD Anderson. Cells were inoculated into the 4th mammary fat pad of 6- to 8-week-old female athymic BALB/c *nu/nu* mice (Department of Experimental Radiation Oncology, MD Anderson) at a density of 5 × 10^6^ in both MCF-7 and ZR-75-1 xenograft models. All mice were implanted with 60-day 17β-estradiol pellets (Innovative Research of America, Sarasota, FL, USA) subcutaneously prior to inoculation of the tumor. Treatments began when tumor diameter reached between 150 and 200 mm^3^. The mice were euthanized at the end point of the study or when tumor diameter reached 1500 mm^3^.

In order to determine in vivo antitumor efficacy of ALRN-6924 in combination with paclitaxel, mice were randomized into 6 groups (vehicle, ALRN-6924 5 mg/kg, ALRN-6924 10 mg/kg, paclitaxel 10 mg/kg, ALRN-6924 5 mg/kg + paclitaxel 10 mg/kg, and ALRN-6924 10 mg/kg + paclitaxel 10 mg/kg, *n* = 6–8 in MCF-7, and *n =* 5 in ZR-75-1 xenograft combination therapy experiment). ALRN-6924 (5, 10 mg/kg) was dosed twice weekly and paclitaxel (10 mg/kg) was dosed weekly, with paclitaxel administered 6 h prior to ALRN-6924. All drugs were administered intravenously via the tail vein over a 26–28-day treatment window.

To evaluate the drug effects and their mechanism of action, we performed pharmacodynamics studies consisting of two additional in vivo experiments. Groups of 4–5 mice with stable tumor sizes ranging between 150 and 200 mm^3^ were randomized into vehicle, ALRN-6924 10 mg/kg, paclitaxel 15 mg/kg, and ALRN-6924 10 mg/kg + paclitaxel 15 mg/kg. Mice were treated for a total of 14 days and euthanized 16 h after the last treatment. Tumors were cut into halves, one half of the tumor was fixed in formalin followed by paraffin embedding, while the other half was snap-frozen.

### Reverse-phase protein arrays

Reverse-phase protein array (RPPA) was conducted by the MD Anderson Functional Proteomics core facility as previously described [25]. Differentially expressed protein association with the canonical pathways was analyzed using the Ingenuity Pathway Analysis (IPA) software (Qiagen, Hilden, Germany).

### Immunohistochemistry

Xenograft tumor samples were formalin-fixed and paraffin-embedded (FFPE). Tumor tissue on hematoxylin and eosin (H&E)-stained slides was confirmed by a board-certified pathologist. Additionally, the presence of tumor necrosis was estimated and given as percentage of the entire tumor.

Immunohistochemistry (IHC) was performed on 4-μm sections using the following antibodies: anti-Ki67 (clone: MIB-1, #M7240, Dako/Agilent, Glostrup, Denmark), anti-cleaved PARP (Asp214) (clone: D64E10, #5625, Cell Signaling Technology, Danvers, USA), anti-p21 (clone: F5, #sc-6246, Santa Cruz, Dallas, USA), anti-p53 (clone: DO-7; Leica Biosystems, Wetzlar, Germany), and anti-TUNEL (ApopTag Peroxidase In Situ Apoptosis Detection Kit, Merck Millipore Sigma, # S7100). For Ki67, p-Histone-H3, and cleaved PARP, the percentage of all positive tumor cells were estimated regardless of staining intensity. The percentage of positive tumor cells and their staining intensity were evaluated for p21 and p53 resulting in an H-score (range 0–300) [26]. TUNEL staining was performed at the Houston Methodist Research Institute (HMRI, Houston, TX, USA). Percentage (%) and density (n/mm^2^) of positive tumor cells were evaluated for TUNEL staining. For digital image analysis, IHC slides were scanned at 20× using Aperio AT2 scanner (Leica Biosystems), and the images were analyzed in HALO software (Indica Labs), using a cytonuclear algorithm. Distinguished necrotic areas and artifacts were excluded. All specimens but one (p21: insufficient staining) could be evaluated for IHC. The IHC evaluation was performed by a board-certified pathologist.

### Bioinformatics

Unsupervised hierarchical cluster analysis was performed in order to generate MCF-7 heat maps of differentially expressed proteins (DEPs) with false discovery rate (FDR) 0.05 (ALRN-6924 vs vehicle and ALRN-6924 plus paclitaxel vs paclitaxel).

### Statistical analysis

For in vitro studies, Student’s *t*-test was carried out for comparison between two groups.

Treatment/control ratio (T/C %) was performed day 27 or day 29, depending on the study by using the formula: (median tumor volume of treatment group/median tumor volume of control) × 100. Antitumor activity was defined as ≤ 40% [27].

Tumor volume (TV) was obtained by using the formula: TV (mm^3^) = ((width)^2^ × length)/2. Change in tumor volume was obtained by using the formula: (TV_DayX_ − TV_Day0_)/TV_Day0_. One-way ANOVA was carried out for in vivo studies in order to determine statistical significance.

## Results

### ALRN-6924 inhibits breast cancer cell growth in vitro

**Fig. 1:**
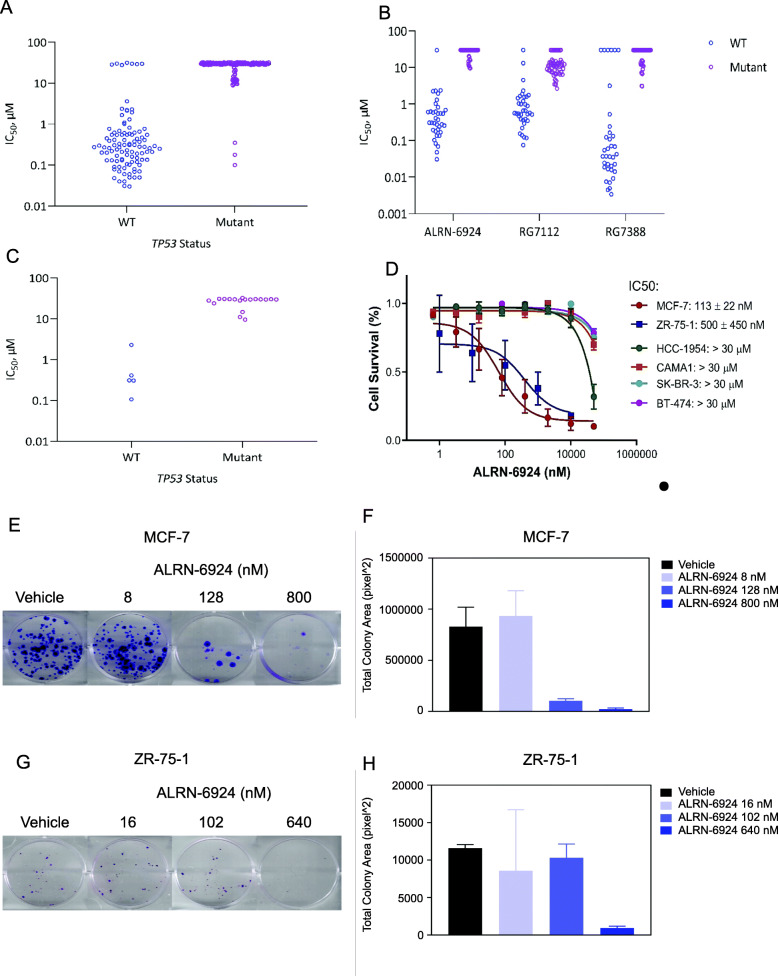
ALRN-6924 anti-proliferative efficacy in vitro*.* **a**–**c** Cell line panel screening. Two cancer cell line panels (Eurofins OncoPanel and focused Horizons Discovery OncoSignature panel) of total 302 cell lines were employed to assess ALRN-6924 activity (**a**). Horizons Discovery OncoSignature panel of 89 cell lines was used to compare three MDM2/MDM4 inhibitors, ALRN-6924, RG7112, and RG7388 (**b**). The effect of ALRN-6924 on breast cancer was assessed in 23 breast cancer cell lines (**c**). For these screening assay (**a**–**c**), cells seeded into 384-well plates overnight were treated the inhibitors at serially diluted concentrations for 72 h. EC_50_ (equally IC_50_, half-maximal inhibitory concentration) was determined using relative cell proliferation rate assessed by changes in nuclear dye uptake or ATP levels. **d** Effect of ALRN-6924 on breast cancer cell lines with different ER status. Cells, seeded in 96-well plates, were treated with ALRN-6924 at serially diluted concentrations for 72 h. Cell proliferation was determined using SRB assay. IC_50_ was calculated using CalcuSyn. Data represent mean ± SD obtained from three replicates. **e**–**h** Colony formation assay. ER+ breast cancer cell lines MCF-7 (**e**, **f**) and ZR-75-1 (**g**, **h**), seeded in 6-well plates, were cultured in the presence of vehicle or ALRN-6924 at different concentrations for 2–3 weeks followed by crystal violet staining. Colony images show representative wells (**e**, **g**). Total colony area (pixel^2) was measured by using NIH ImageJ software (**f**, **h**). Data represent mean ± SD from triplicates

In order to confirm the anti-cancer activity of ALRN-6924 is associated with functional p53, we tested the efficacy of ALRN-6924 in 302 cell lines which represent multiple solid and hematologic cancer types. We found that ALRN-6924 was highly active in 95 cell lines out of 102 cell lines with WT *TP53*. IC_50_s of ALRN-6924 in most of these *TP53*-WT cell lines were below 1 μM. In contrast, from 200 cell lines that carry mutant or null *TP53*, 197 cell lines were resistant to the drug with IC_50_s over 10–30 μM (Fig. 1a, Supplementary Table 1). ALRN-6924 was also evaluated in 89 cell lines side-by-side with two other well-studied MDM2 inhibitors: RG7112, an orally administered small molecule that reached phase 1 clinical studies, and RG7388 (idasanutlin), a next-generation MDM2 inhibitor [28, 29]. As shown in Fig. 1b, all three agents show superior activity in *TP53*-WT cell lines than mutant ones (Supplementary Table 1). Among the cancer cell lines included in the screening panel shown in Fig. 1a, 23 were breast cancer cell lines. Of these, 5 WT *TP53* cell lines were sensitive to ALRN-6924 with a median IC_50_ of 0.31 nM, while the other 18 cell lines with mutant *TP53* were resistant with IC_50_s over 30 μM (Fig. 1c, Supplementary Table 1).

We also determined the anti-proliferative activity of ALRN-6924 on ER+ breast cancer cells in an independent experiment. ALRN-6924 demonstrated potent anti-proliferative activity in a dose-dependent manner in two ER+ cell lines with WT *TP53*, MCF-7 and ZR-75-1, with IC_50_ value of 113 nM and 500 nM respectively (Fig. 1d). On the other hand, four ER+ cell lines with mutant *TP53*, HCC-1954, CAMA1, SK-BR-3, and BT-474, showed resistance to ALRN-6924 with average IC_50_ over 30 μM (Fig. 1d). The effect of the agent was further demonstrated in colony formation assays where ALRN-6924 was able to decrease the clonogenic capacity of both MCF-7 and ZR-75-1 in a dose-dependent manner (Fig. 1e–h).

### ALRN-6924 demonstrates synergistic activity with both paclitaxel and eribulin in vitro

**Fig. 2:**
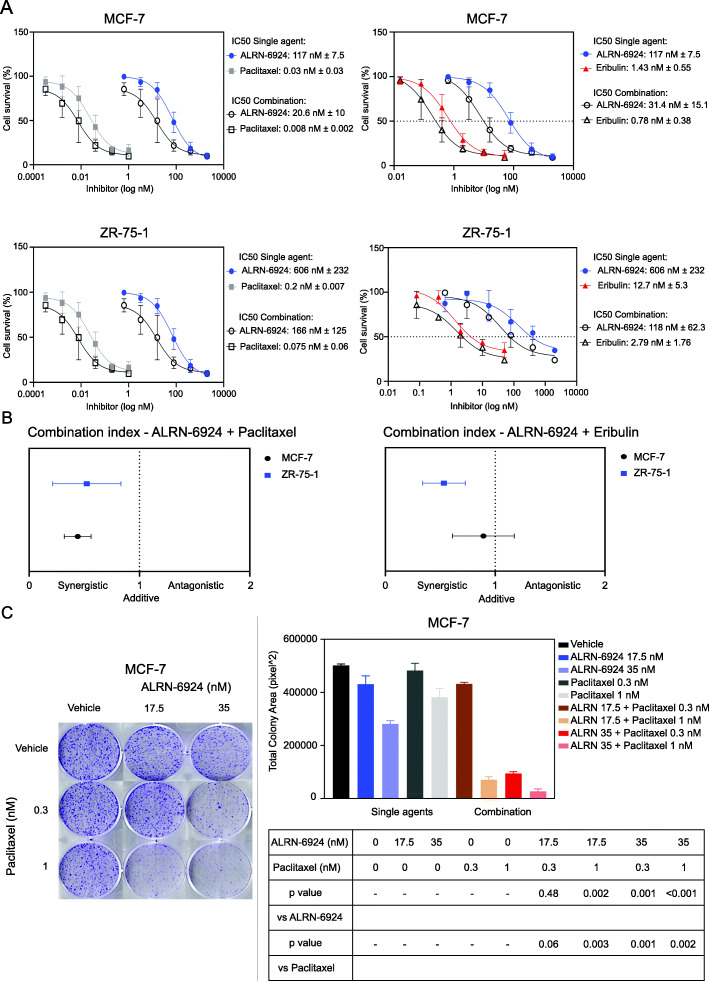
The combination of ALRN-6924 and chemotherapeutic agents synergistically inhibit cell proliferation in vitro*.* **a** Cells were seeded in 96-well plates at a density of 5000–10,000 cells per well then treated for 72 h with vehicle, ALRN-6924, paclitaxel, eribulin, or combination of each. Effect on cell growth was determined using the SRB assay. IC_50_ values were calculated using GraphPad Prism software. **b** Combination indices were calculated using the Chou-Talalay method with a CI < 1 indicating synergistic activity. **c** MCF-7 colony formation assay treated for 14 days with either vehicle, ALRN-6924, paclitaxel, or combination of ALRN-6924 and paclitaxel. Total colony area was measured by using NIH ImageJ software. Mean ± SD (*n* = 2) values are shown

We hypothesized that combining ALRN-6924 with chemotherapeutic agents paclitaxel or eribulin would enhance the antitumor efficacy of either single agent. Indeed, this was evident in both cell lines. The combination of ALRN-6924 with paclitaxel or eribulin mutually benefited each other and demonstrated lower IC_50_ and synergistic activity (CI < 1) compared to single agent treatment alone in both cell lines (Fig. 2a, b). Next, we assessed the clonogenic capacity of the cell lines in the presence of either single agent or combination of drugs with colony formation assay. MCF-7 cells were treated with vehicle control, ALRN-6924, paclitaxel or eribulin, or the combination of ALRN-6924 with paclitaxel or eribulin. We found that the clonogenic capacity was significantly reduced in the presence of ALRN-6924 combined with paclitaxel or eribulin compared to either single agent (Fig. 2c, S1).

### ALRN-6924 combined with paclitaxel induces cell cycle arrest and apoptosis

**Fig. 3:**
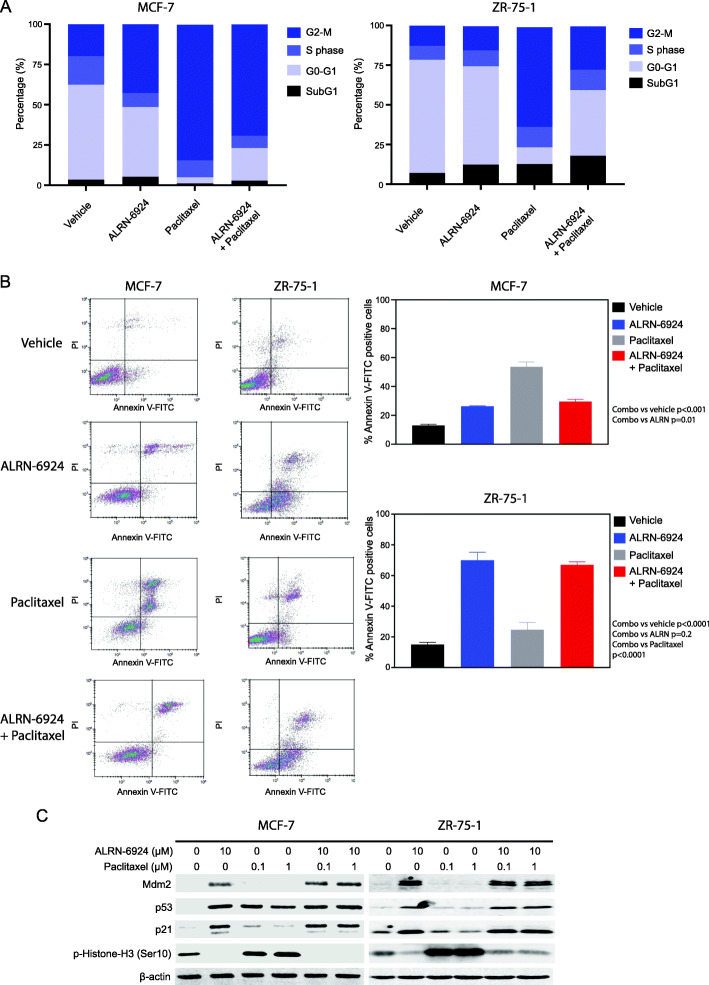
ALRN-6924 combined with paclitaxel reactivates p53 and induces cell cycle arrest and apoptosis in vitro. **a** Cell cycle analysis was carried out by flow cytometry after 24-h treatment with vehicle, ALRN-6924 (10 μM), paclitaxel (1 μM), or combination of both. Percentages of cells in Sub-G1, G0-G1, S, and G2-M phases of cell cycle are depicted. **b** Flow cytometry analysis of Annexin V-positive cells after treatment for 72 h by vehicle, ALRN-6924 (10 μM), paclitaxel (1 μM), or combination of both in both MCF-7 and ZR-75-1. Mean ± SD (*n* = 3) values are shown. **c** Immunoblotting of p53, p21, Mdm2, and p-Histone-H3 (Ser10). MCF-7 and ZR-75 were treated with vehicle control, ALRN-6924 at 10 μM, or paclitaxel 0.1, 1 μM, or combination of both for 24 h

We next explored the mechanistic basis of synergy noted for the combination of ALRN-6924 and paclitaxel by flow cytometry. In MCF-7 cells, we found indirect signs of mitotic crisis with an increase in percentage of cells in the G2-M phase in all the treatment groups, compared to the vehicle control (Fig. 3a). However, we were not able to detect a statistical significance in G2-M cell changes between the single agent groups and combination group. We also observed reduced cell population in the S phase in both combination and single agent ALRN-6924 treatment arms (Fig. 3a). In ZR-75-1 cells, we noticed an increase in G2-M cells in single agent paclitaxel and combination groups. However, cell portions in the S phase were not significantly altered in the treatment groups compared to the vehicle control (Fig. 3a).

Treatment for 72 h with ALRN-6924 combined with paclitaxel resulted in a higher rate of apoptosis compared to vehicle or with paclitaxel alone in ZR-75-1 cells (*p* < 0.0001), but this was not enhanced compared to the effect of ALRN-6924 monotherapy. Apoptosis was also enhanced with the combination compared to vehicle in MCF-7 cells, but this was not greater than observed in paclitaxel alone (Fig. 3b).

### Influence of ALRN-6924 combined with paclitaxel on p53 pathway and mitosis

We next evaluated the effects of ALRN-6924 combined with paclitaxel on p53 pathway expression and mitosis in both cell lines. Cells were treated with vehicle, ALRN-6924, paclitaxel, or combination of both for 24 h; lysed; and immunoblotted. We found that both ALRN-6924 and paclitaxel single agent treatments at the indicated doses were capable of increasing p53 levels in both cell lines (paclitaxel had less effect in ZR-75-1 cells) compared to the vehicle control (Fig. 3c). Consequently, we looked at the impact of drug treatments on the expression of p21 and MDM2, the downstream effectors of p53. We found that ALRN-6924, but not paclitaxel, also increased p21 and Mdm2 levels in both cell lines (Fig. 3c). However, we did not detect further enhancement in the expression of all these markers by combination treatments (Fig. 3c).

Phosphorylation of Histone H3 (p-HH3) at Ser10 residue is a cell cycle marker of the G2-M phase and is associated with chromosome condensation during mitosis [30]. Interestingly, we found that ALRN-6924 single agent treatment substantially decreased the levels of p-HH3, while in contrast paclitaxel showed an enhancing effect on this marker in both cell lines (Fig. 3c). Because of their opposing effects on p-HH3, we did not observe any combinatorial effect on this marker (Fig. 3c).

### ALRN-6924 mutually enhances both paclitaxel and eribulin antitumor efficacy and inhibits tumor growth in vivo

**Fig. 4:**
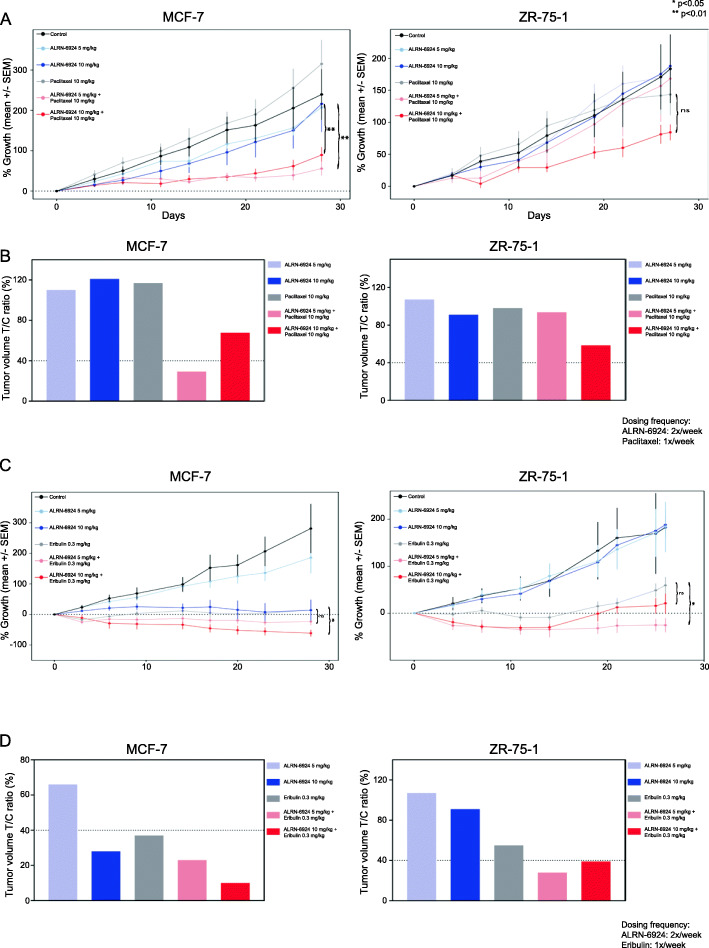
ALRN-6924 in combination with chemotherapeutic agents inhibits in vivo tumor growth. **a** Female athymic nu/nu mice bearing MCF-7 and ZR-75-1 xenografts were treated with vehicle, ALRN-6924 5 mg/kg or 10 mg/kg, paclitaxel 10 mg/kg, or the combination of each. All treatments were administered intravenously at the indicated times. Depicted values represent mean ± SEM (*n* = 5–8). Statistical significance was determined using one-way analysis of variance (ANOVA) analysis of the rate of change in tumor volume. **b** Using median tumor volume, T/C ratios were obtained. Efficacy defined as < 40% T/C ratio at the end of the studies (28 days for MCF-7 and 26 days for ZR-75-1). **c** Female athymic nu/nu mice bearing MCF-7 and ZR-75-1 xenografts were treated with vehicle, ALRN-6924 5 mg/kg or 10 mg/kg, eribulin 0.3 mg/kg, or the combination of each. All treatments were administered intravenously at the indicated times. Depicted values represent mean ± SEM (*n* = 5–8). **d** Using median tumor volume, T/C ratios were obtained

The in vivo antitumor efficacy of ALRN-6924 combined with paclitaxel or eribulin was evaluated in ZR-75-1 and MCF-7 xenografts. In MCF-7 xenografts, combinations of ALRN-6924 (5 and 10 mg/kg) with paclitaxel (10 mg/kg) and combination of ALRN-6924 (10 mg/kg) with eribulin (0.3 mg/kg) effectively inhibited tumor growth and had superior antitumor efficacy compared to single agents alone (*p* = 0.003, *p* = 0.005, and *p* = 0.03 respectively) (Fig. 4a, c). Analysis of treatment/control ratio of tumor volume (T/C %) of both ALRN-6924 plus paclitaxel and eribulin at the end of the study (day 28) revealed significantly reduced T/C ratios in all combinatorial therapies except ALRN-6924 (10 mg/kg) plus paclitaxel (10 mg/kg) (Fig. 4b, d).

In the ZR-75-1 model, the decrease in tumor growth when combining ALRN-6924 with paclitaxel compared to monotherapy was not statistically significant (Fig. 4a, b). However, when ALRN-6924 (5 mg/kg) was combined with eribulin (0.3 mg/kg), it demonstrated superior antitumor efficacy compared to either agent alone (*p* = 0.01) (Fig. 4c). This was further evidenced by efficacious T/C ratio in ZR-75-1 xenografts at the end of the study (day 26) (Fig. 4d).

### ALRN-6924 combined with paclitaxel decreases proliferation, arrests cell cycle by reactivating p53, and induces apoptosis in vivo

**Fig. 5:**
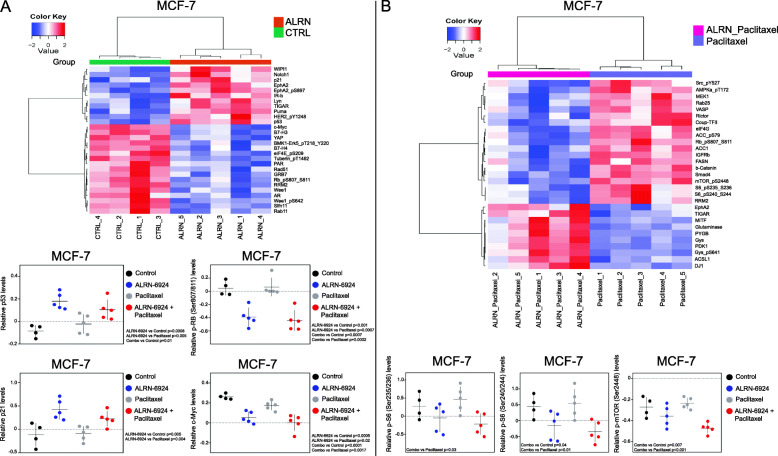
Functional proteomics of MCF-7 xenograft demonstrating in vivo effects of ALRN-6924 combined with paclitaxel on proliferative markers and cell cycle regulators. **a** Heat map demonstrating 28 DEPs (ALRN-6924 vs vehicle control) which was generated by unsupervised hierarchical cluster analysis of RPPA obtained from pharmacodynamics studies. Scatter plots based on RPPA data of MCF-7 pharmacodynamics study depicting in vivo p53, p21, c-Myc, and p-Rb activity. Mean ± SD is shown (*n* = 4–5). **b** Heat map showing 29 DEPs (ALRN+paclitaxel vs paclitaxel). Scatter plots demonstrating in vivo mTOR pathway activity after vehicle, ALRN-6924, paclitaxel, or combination of both treatments. Mean ± SD (*n* = 4–5) is shown

**Fig. 6:**
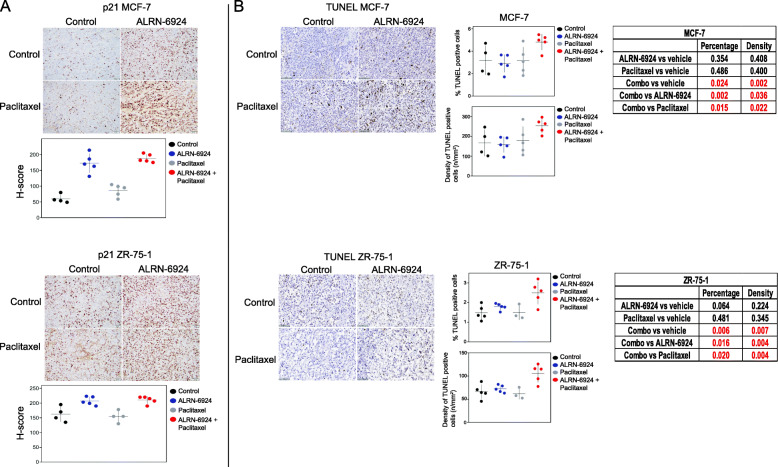
Immunohistochemistry demonstrating in vivo cell cycle arrest through p21 and enhanced apoptotic rate when combining ALRN-6924 with paclitaxel. **a** Scatter plots of in vivo immunohistochemistry (IHC) H-score p21 activity in both MCF-7 and ZR-75-1 xenografts. Mean ± SD is shown. **b** IHC images and scatter plots of in vivo percentages and density of TUNEL-positive tumor cells after treatment with vehicle, ALRN-6924, paclitaxel, or combination of both in both MCF-7 and ZR-75-1 xenografts. Mean ± SD is shown

We next performed an in vivo pharmacodynamics study to explore the mechanisms of action. Expression of proteins extracted from xenograft tumor tissues treated with ALRN-6924, paclitaxel, and their combination were profiled by RPPA. Heat maps generated from RPPA data demonstrated 28 DEPs when comparing ALRN-6924 to vehicle control (Fig. 5a). RPPA additionally revealed enhanced reactivation of p53, and its downstream effector, p21 in both single agent ALRN-6924 and combination arms (Fig. 5a). Combination versus paclitaxel analysis revealed 29 DEPs (Fig. 5b). Dephosphorylation of retinoblastoma (Rb) protein at Serine 807 and 811 residues have previously been shown to trigger apoptosis [31]. In our studies, we observed a robust downregulation of p-Rb (Ser807/811) when combining ALRN-6924 with paclitaxel compared to vehicle and paclitaxel alone. Markers of pathways involved in cell proliferation such as the mechanistic target of rapamycin (mTOR) [32] and c-Myc oncoprotein [33] were additionally found to be decreased in the combination arm compared to vehicle or either single agent, explaining possibly off-target anti-proliferative effects when combining ALRN-6924 with paclitaxel (Fig. 5b). IHC H-score analysis demonstrated a robust increase in p21 expression in the combination arm of both xenograft models which is consistent with the RPPA find above (Fig. 6a). IPA analysis demonstrated p53 signaling pathway as one of the top canonical pathways involved (Supplementary Table 1).

To validate these proteomic changes, we performed an immunoblotting assay. The results showed that single ALRN-6924 treatment dose-dependently increased the expression levels of p53 and p21 (Fig. S2A, B, and C), but decreased c-Myc and p-Rb levels (Fig. S2A, D, and E). We also noticed that ALRN-6924 dose-dependently reduced the ratio of LC3-I/LC3-II which are both involved in autophagy (Fig. S2A, F, and G) [34]. ALRN-6924 had a moderate decreasing effect on p-S6, which is a downstream effector of mTOR and controls protein synthesis [35] (Fig. S2A, and H). On the other hand, paclitaxel alone at high doses enhanced p53 levels but reduced c-Myc and p-S6 levels (Fig. S2A, B, D, and H). There were no substantial changes in expression levels of p21, p-Rb, and LC3 ratio in the paclitaxel groups (Fig. S2A, C, E, F, and G). The enhanced combinatorial efficacy on p-Rb, p-S6, and c-Myc was not seen in ALRN-6924 combination with paclitaxel in vitro (Fig. S3A and D). In the combination assay, we found that compared to the single drug treatment and vehicle control, combination of ALRN-6924 at doses of 50, 100, and 500 nM with paclitaxel at doses 1 and 10 μM further enhanced expression of both p53 and p21 (Fig. S3A, right six lanes, B, and C). The combination at higher doses also had a strengthened inhibitory effect on the LC3 ratio (Fig. S3A, and E). Taken together, these immunoblotting results correlate with the proteomic findings in RPPA.

To evaluate in vivo apoptosis, we performed TUNEL staining. The results revealed a significantly higher percentage and density of TUNEL-positive tumor cells in the combination arm compared to either single agent alone in both xenograft models (Fig. 6b).

## Discussion

The majority of human tumors have a mutated *TP53* gene. We have previously shown that almost a third of patients with metastatic hormone receptor-positive breast cancer have *TP53* mutations, and *TP53* mutations were associated with a shorter recurrence-free survival and overall survival [36], demonstrating the importance of p53 pathway. However, most patients with hormone receptor-positive breast cancers still have WT *TP53* status [2]. Many breast cancers exhibit *Mdm2/Mdm4* amplifications; however, these alterations often do not co-occur with *TP53* mutations [37]. Interestingly, MDM2 overexpression was found to induce chemoresistance and additionally enhances invasiveness and motility of breast cancer cells [5]. Based on this knowledge, it provided us a therapeutic rationale to analyze a possible cancer therapeutic target by itself and in combination with standard chemotherapeutic agents in breast cancer models. Our findings revealed the targeted agent ALRN-6924 was able to enhance the antitumor efficacy of both paclitaxel and eribulin in vitro and in vivo. We were also able to demonstrate that both ALRN-6924 alone and in combination with paclitaxel restore p53 activity and inhibit progression of cell cycle mainly through the activity of p21.

We demonstrated enhanced induction of apoptosis in vivo but not in vitro. This could potentially be explained by the effect of ALRN-6924 combined with paclitaxel on the stromal elements present in xenograft models. p-HH3 marker has emerged as a proliferative or mitotic marker compared to other proliferative markers such as Ki67, Mitotic Activity Index (MAI), and demonstrated greater accuracy [38]. p-HH3 levels were lower in both single agent ALRN-6924 and combination cohorts compared to vehicle, suggesting that ALRN-6924 possibly is not acting through mitosis, but rather through DNA damage [39]. The increased p-HH3 levels observed with single agent paclitaxel has previously been described when cells are exposed to noxious stimuli and subsequent impending apoptotic event [40, 41]. There was a response to ALRN-6924 single agent on cell proliferation and apoptosis in ZR-75-1 cells. Cell proliferation and apoptosis involve different mechanisms. Thus, the apoptosis pathway in ZR-75-1 cells could be more vulnerable than the proliferation pathway, compared to MCF-7 cells. However, this warrants future investigation. Several molecular markers of autophagy have been studied to date, but the conversion of LC3-I to LC3-II has been accepted as the gold standard for autophagosome formation. Upon autophagic signal, the cytosolic LC3-I is conjugated to phosphatidylethanolamine (PE) to form LC3-PE conjugate (LC3-II), which is recruited to the autophagosomal membranes. Thus, the reduced ratio of LC3-I/LC3-II in this paper represents activation of autophagy by MDM2/X inhibition. MDM2 interacts with pRb which forms a triplex complex with p53. Our RPPA data showed a co-regulation of p53 and pRb by the MDM2 inhibitor (Fig. 5a). MDM2 is also known to have p53-independent actions. MDM2 promotes cell proliferation by activating NFκB pathway but inducing degradation of tumor suppressor FoxO3A which acts via ERK. MDM2 also downregulates E-Cadherin, leading to invasion. However, we did identify the changes in these markers in our RPPA study (Fig. 5a). Therefore, the p53-independent roles may not be primary roles of MDM2 in these cell lines.

In a separate study, we examined the target specificity of MDM2/X inhibitors. We created MDMX knockout cell lines in OCI-AML3 cells (Fig. S4A, B). The data showed that MDMX KO cells were more sensitive to MDM2/X inhibitor ALRN-6924 and MDM2 inhibitor RG7388 than WT cells (Fig. S4C, D). However, further work is needed to dissect the role of MDMX inhibition in ALRN-6924’s antitumor efficacy, and how this efficacy profile differs from those of MDM2 inhibitors. Interestingly, clinical trials appear to also demonstrate differences in toxicity profile, as ALRN-6924 appears to have less bone marrow toxicity noted in phase I trials compared to what has been reported with other MDM2 inhibitors [42, 43].

Another question we have not specifically addressed is the effect of ALRN-6924 on the tumor microenvironment. Cell line-derived xenografts have mouse stroma. They are not the optimal model to assess the effect of ALRN-6924 on the tumor microenvironment. Further, our RPPA assay has been optimized to detect human markers and is focused on tumor markers, and thus, our current studies are not conducive to address this question. Therefore, we plan to test the effect of ALRN-6924 on the tumor microenvironment in ongoing clinical trials.

mTOR pathway is one of the classical canonical pathways involved in cancer cell proliferation and metastasis [44]. Emerging evidence has demonstrated that the mTOR pathway has been associated with endocrine therapy resistance [45], especially in metastatic breast cancers [46]. By inhibiting this pathway, it has been shown to prevent resistance and restore sensitivity in hormone-positive breast cancers [47]. Clinically, mTOR inhibitor everolimus is known to increase progression-free survival when given in combination with exemestane compared to exemestane alone in post-menopausal women with advanced hormone receptor-positive breast cancer [48]; thus, mTOR is a proven target in breast cancer. Our study revealed an off-target effect of ALRN-6924 combined with paclitaxel, downregulating p-mTOR and p-S6, possibly explaining one of the potential mechanisms of decreased proliferation when combining these two drugs in vivo.

Additional mechanistic studies are required in order to explore and determine the interplay between ALRN-6924 and paclitaxel. The question also remains on how the combination of ALRN-6924 and paclitaxel or eribulin affect other additional p53-dependent and independent pathways, potentially revealing additional affected pathways.

Changes in a single residue by hot-spot mutations can impair p53 activity which transforms p53 into a gain-of-function oncoprotein [49]. Two scenarios have been demonstrated where both *TP53 WT* and mutant p53 (*TP53 MT*) can affect each other, one being “dominant-positive” effect, where *TP53 WT* activity suppresses *TP53 MT* activity, or “dominant-negative effect” where *TP53 MT* allele inactivates *TP53 WT* allele [50]. We did not test the combination of ALRN-6924 and paclitaxel or eribulin in a setting with both *TP53 MT* and *TP53 WT*; further studies are needed in order to determine if this combination can rescue *TP53 WT* and suppress mutant p53 activity in order to enhance paclitaxel or eribulin sensitivity.

Notably, our studies have been conducted in immunocompromised mice, not allowing us to assess the immune effects of Mdm4/Mdm2 inhibition. Recently, there have been anectodal reports of hyperprogression on immunotherapy in patients with *Mdm2* amplification [51]. The role of p53 in signal transduction pathways involved in the regulation of the immune response has been extensively discussed [52, 53]. In murine syngeneic models, ALRN-6924 and selective Mdm2 antagonists were shown to have co-stimulatory activity in T cells and increase PD-L1 expression, and in combination with anti-PD1 and anti-PDL1, demonstrated enhanced antitumor activity not only in *TP53 WT* but also in *TP53 MT* tumors [54]. Thus, further exploration is needed to determine whether the antitumor efficacy of ALRN-6924 combination with chemotherapy would be further enhanced in immunocompetent models.

## Conclusions

A large portion of hormone receptor-positive breast cancer have *TP53 WT* status. Reactivating p53 in order to enhance antitumor efficacy of chemotherapy is an attractive option. Mdm4 and Mdm2 proteins known to regulate *TP53* levels are frequently elevated in hormone receptor-positive breast cancer. In this study, we demonstrated the antitumor efficacy of ALRN-6924, a dual inhibitor of Mdm4 and Mdm2, in ER+ breast cancer cell line models and identified significant synergy with combination of ALRN-6924 with chemotherapeutic agents. These results support the clinical trial of ALRN-6924 in combination with paclitaxel (ClinicalTrials.gov identifier: NCT03725436) in patients with advanced, metastatic, or unresectable solid tumors.

## Supplementary Information

**Additional file 1: Supplementary Figure 1.** ALRN-6924 combined with eribulin synergistically inhibit clonogenic capacity of MCF-7 breast cancer cell line. MCF-7 colony formation assay treated with either vehicle, ALRN-6924, paclitaxel, or combination of both. Total colony area was measured by using NIH ImageJ software. Mean +/- SD is demonstrated. **Supplementary Figure 2.** In vitro validation of in vivo functional proteomic changes after treatment with ALRN-6924 and paclitaxel. A. MCF-7 cells were treated with vehicle, ALRN-6924 or paclitaxel in a dose-dependent fashion for 24 hours followed by immunoblotting with indicated antibodies. B. Quantification of relative p53 levels by standardizing to β-actin. C. Quantification of relative p21 levels by standardizing to β-actin. D. Quantification of relative c-Myc levels by standardizing to β-actin. E. Quantification of relative p-Rb (Ser807/811) levels by standardizing to β-actin. F. Quantification of relative LC3-I/LC3-II ratio levels by standardizing to β-actin. G. Quantification of relative LC3 levels by standardizing to β-actin. H. Quantification of relative p-S6 (Ser235/236) levels by standardizing to β-actin. **Supplementary Figure 3.** Immunoblotting of signaling pathways affected by ALRN-6924 combined with paclitaxel. **A.** MCF-7 cells were treated with vehicle, ALRN-6924, paclitaxel or combination of both in a dose-dependent fashion for 24 hours followed by immunoblotting with indicated antibodies. B. Quantification of relative p53 levels by standardizing to β-actin. C. Quantification of relative p21 levels by standardizing to β-actin. D. Quantification of relative p-Rb (Ser807/811) levels by standardizing to β-actin. E. Quantification of relative LC3-I/LC3-II ratio levels by standardizing to β-actin. **Supplementary Figure 4.** MDMX knockout cell line models. A. Immunoblotting of MDMX. Two MDMX knockout clones (M1 and M4, equally KO-1 and KO-2) were established in Myeloid leukemia cell line OCI-AML3 cells. MDMX was blotted with anti-MDMX antibody. B. Cell proliferation assay. Cell proliferation of two MDMX knockout cell line clones and control cell line was examined using the same methods as described in Fig. 1. C-D. Drug sensitivity assay. Two MDMX knockout cell line clones and control cell line were treated ALRN-6924 (C) and RG7388 (D) to assess the efficacy of these drugs using the same method as described in Fig. 1. **Supplementary Table 1.** Cell line panels for drug screening. **Supplementary Table 2.** IPA analysis of MCF-7 ALRN-6924 + Paclitaxel vs Control.

## Data Availability

The datasets during and/or analyzed during the current study are available from the corresponding author on reasonable request.
