# Supplementary material for: First in class dual MDM2/MDMX inhibitor ALRN-6924 enhances antitumor efficacy of chemotherapy in TP53 wild-type hormone receptor-positive breast cancer models
Source: Breast Cancer Res. 2021 Mar 4;23:29. doi: 10.1186/s13058-021-01406-x (PMC7934277; doi:10.1186/s13058-021-01406-x)
Supplement: Supplementary file 1 — Additional file 1: Supplementary Figure 1. ALRN-6924 combined with eribulin synergistically inhibit clonogenic capacity of MCF-7 breast cancer cell line. MCF-7 colony formation assay treated with either vehicle, ALRN-6924, paclitaxel, or combination of both. Total colony area was measured by using NIH ImageJ software. Mean +/- SD is demonstrated. Supplementary Figure 2. In vitro validation of in vivo functional proteomic changes after treatment with ALRN-6924 and paclitaxel. A. MCF-7 cells were treated with vehicle, ALRN-6924 or paclitaxel in a dose-dependent fashion for 24 hours followed by immunoblotting with indicated antibodies. B. Quantification of relative p53 levels by standardizing to β-actin. C. Quantification of relative p21 levels by standardizing to β-actin. D. Quantification of relative c-Myc levels by standardizing to β-actin. E. Quantification of relative p-Rb (Ser807/811) levels by standardizing to β-actin. F. Quantification of relative LC3-I/LC3-II ratio levels by standardizing to β-actin. G. Quantification of relative LC3 levels by standardizing to β-actin. H. Quantification of relative p-S6 (Ser235/236) levels by standardizing to β-actin. Supplementary Figure 3. Immunoblotting of signaling pathways affected by ALRN-6924 combined with paclitaxel. A. MCF-7 cells were treated with vehicle, ALRN-6924, paclitaxel or combination of both in a dose-dependent fashion for 24 hours followed by immunoblotting with indicated antibodies. B. Quantification of relative p53 levels by standardizing to β-actin. C. Quantification of relative p21 levels by standardizing to β-actin. D. Quantification of relative p-Rb (Ser807/811) levels by standardizing to β-actin. E. Quantification of relative LC3-I/LC3-II ratio levels by standardizing to β-actin. Supplementary Figure 4. MDMX knockout cell line models. A. Immunoblotting of MDMX. Two MDMX knockout clones (M1 and M4, equally KO-1 and KO-2) were established in Myeloid leukemia cell line OCI-AML3 cells. MDMX was blotted w [file 13058_2021_1406_MOESM1_ESM.pdf]

Figure S1

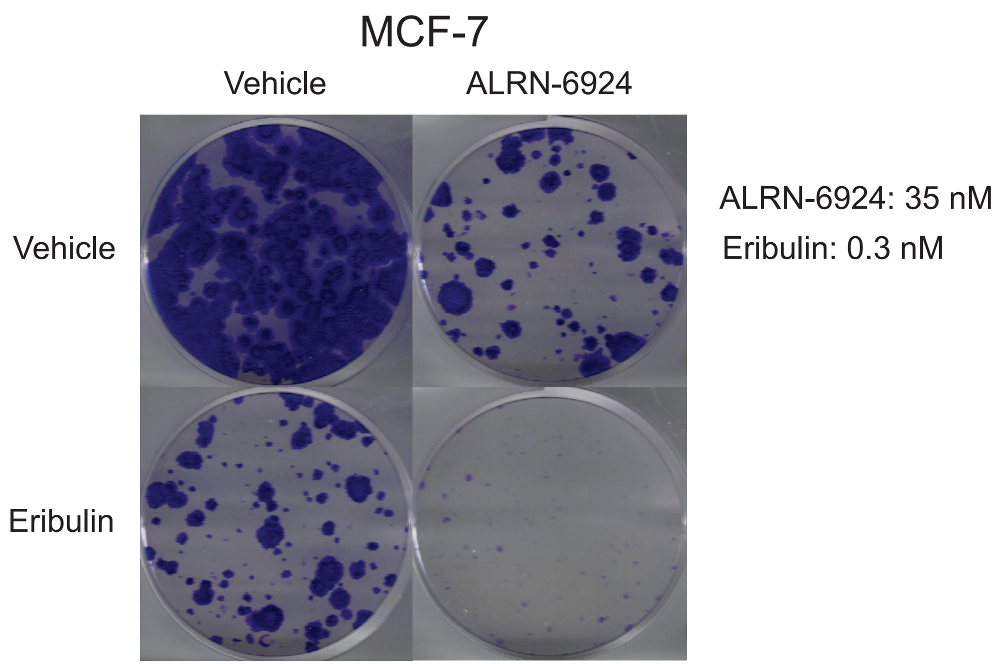
MCF-7
Vehicle
ALRN-6924
Vehicle
Eribulin
ALRN-6924: 35 nM
Eribulin: 0.3 nM

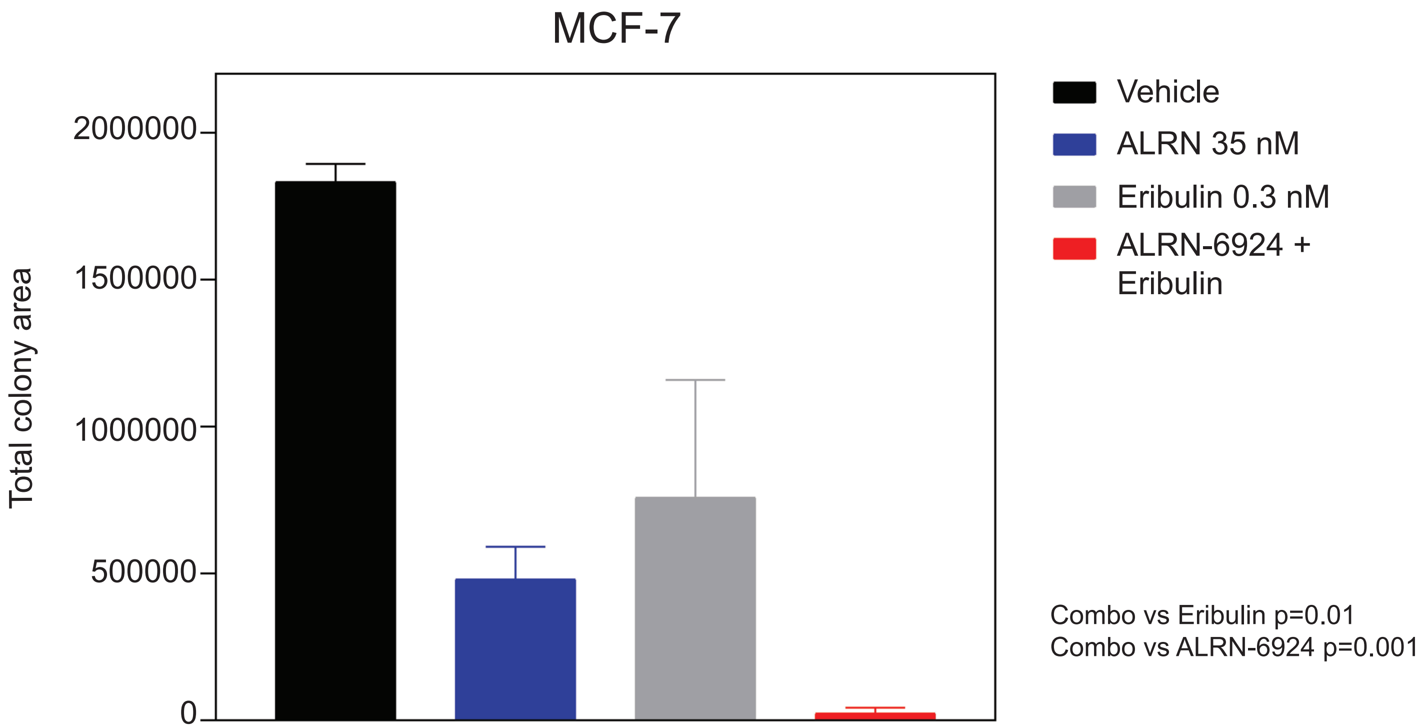
MCF-7
Total colony area
2000000
1500000
1000000
500000
0
Vehicle
ALRN 35 nM
Eribulin 0.3 nM
ALRN-6924 + Eribulin
Combo vs Eribulin p=0.01
Combo vs ALRN-6924 p=0.001

Figure S2

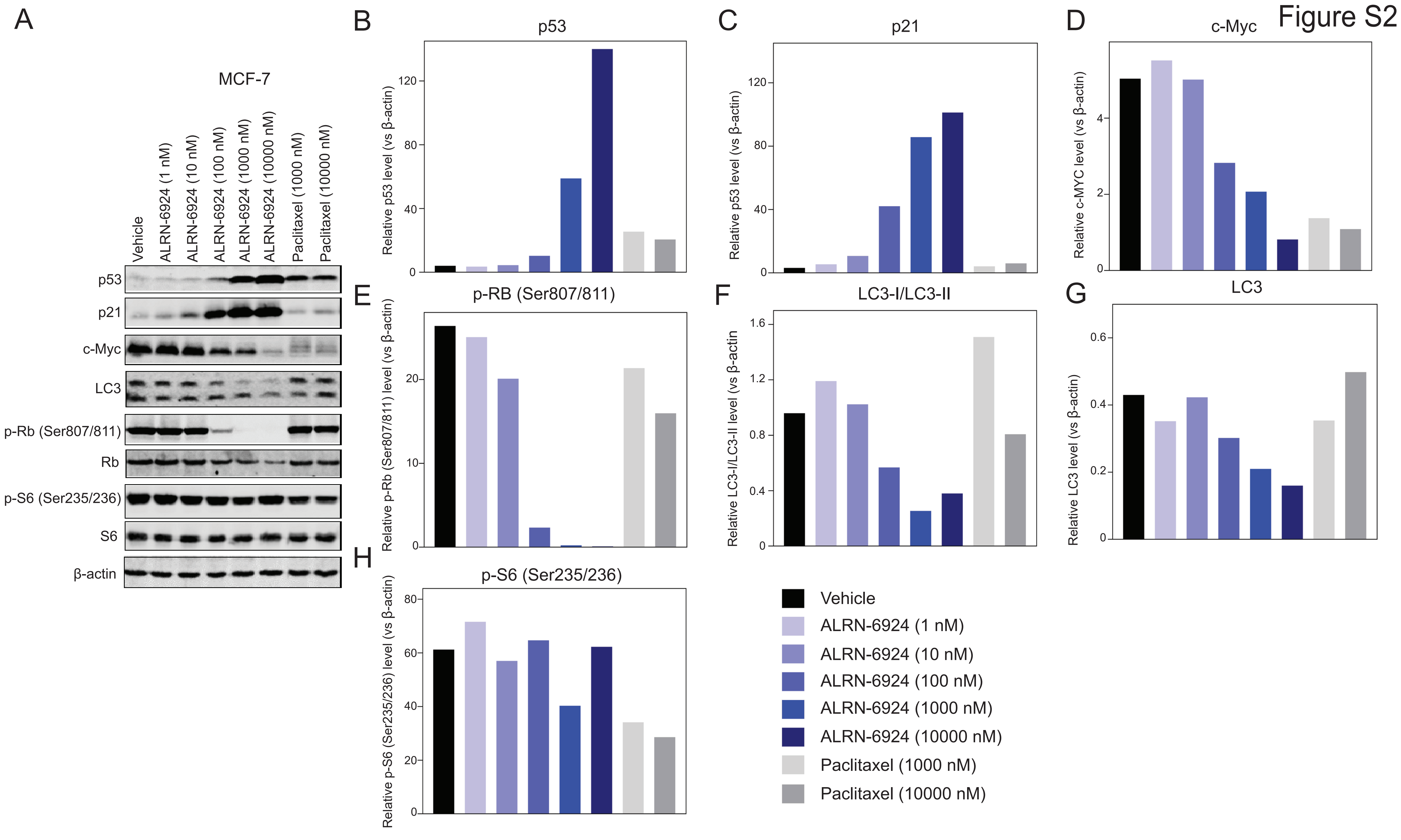

A
MCF-7
Vehicle
ALRN-6924 (1 nM)
ALRN-6924 (10 nM)
ALRN-6924 (100 nM)
ALRN-6924 (1000 nM)
ALRN-6924 (10000 nM)
Paclitaxel (1000 nM)
Paclitaxel (10000 nM)
p53
p21
c-Myc
LC3
p-Rb (Ser807/811)
Rb
p-S6 (Ser235/236)
S6
β-actin
B
p53
Relative p53 level (vs β-actin)
C
p21
Relative p53 level (vs β-actin)
D
c-Myc
Relative c-MYC level (vs β-actin)
E
p-RB (Ser807/811)
Relative p-Rb (Ser807/811) level (vs β-actin)
F
LC3-I/LC3-II
Relative LC3-I/LC3-II level (vs β-actin
G
LC3
Relative LC3 level (vs β-actin)
H
p-S6 (Ser235/236)
Relative p-S6 (Ser235/236) level (vs β-actin)
Vehicle
ALRN-6924 (1 nM)
ALRN-6924 (10 nM)
ALRN-6924 (100 nM)
ALRN-6924 (1000 nM)
ALRN-6924 (10000 nM)
Paclitaxel (1000 nM)
Paclitaxel (10000 nM)

Figure S3

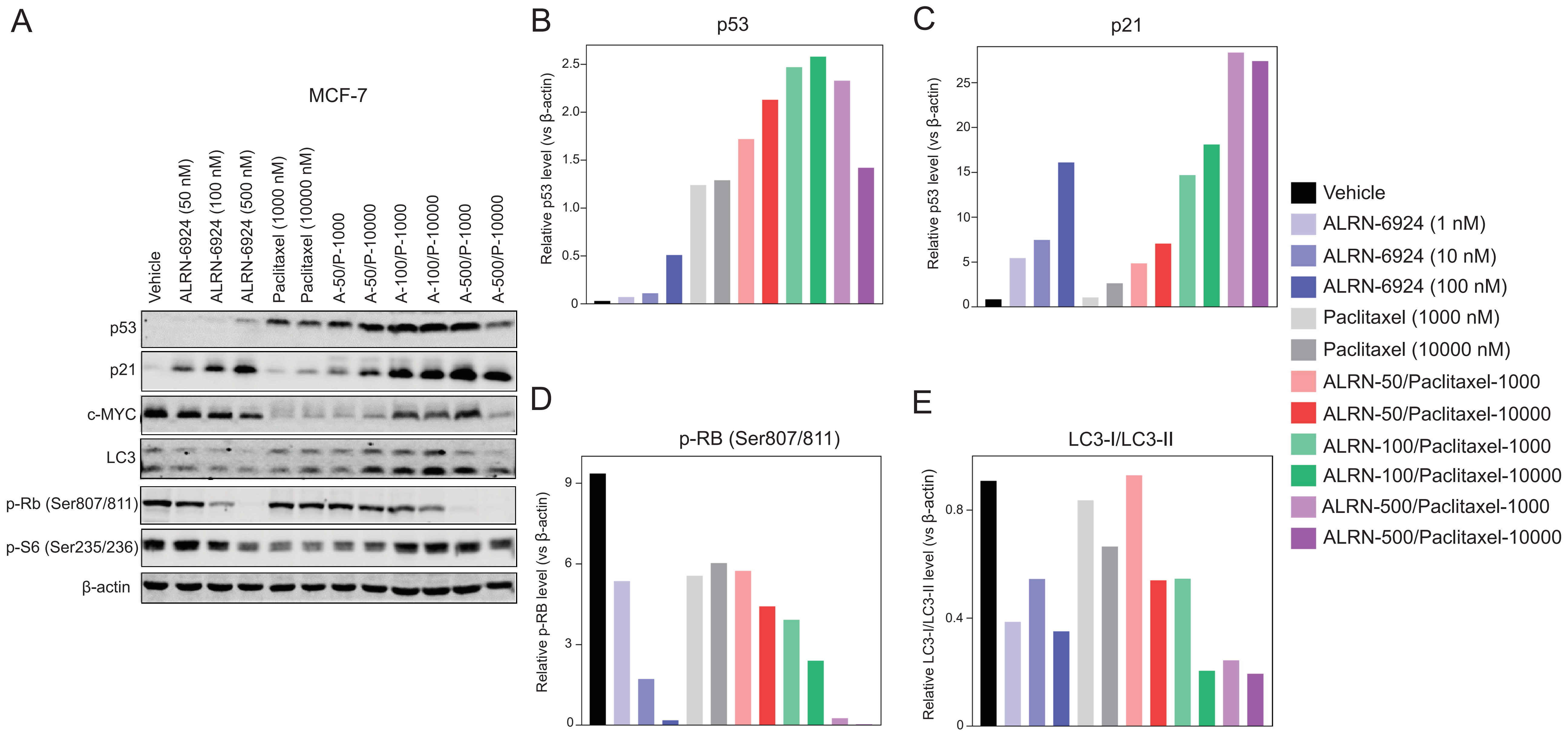

A
MCF-7
Vehicle
ALRN-6924 (50 nM)
ALRN-6924 (100 nM)
ALRN-6924 (500 nM)
Paclitaxel (1000 nM)
Paclitaxel (10000 nM)
A-50/P-1000
A-50/P-10000
A-100/P-1000
A-100/P-10000
A-500/P-1000
A-500/P-10000
p53
p21
c-MYC
LC3
p-Rb (Ser807/811)
p-S6 (Ser235/236)
β-actin
B
p53
Relative p53 level (vs β-actin)
C
p21
Relative p53 level (vs β-actin)
D
p-RB (Ser807/811)
Relative p-RB level (vs β-actin)
E
LC3-I/LC3-II
Relative LC3-I/LC3-II level (vs β-actin)
Vehicle
ALRN-6924 (1 nM)
ALRN-6924 (10 nM)
ALRN-6924 (100 nM)
Paclitaxel (1000 nM)
Paclitaxel (10000 nM)
ALRN-50/Paclitaxel-1000
ALRN-50/Paclitaxel-10000
ALRN-100/Paclitaxel-1000
ALRN-100/Paclitaxel-10000
ALRN-500/Paclitaxel-1000
ALRN-500/Paclitaxel-10000

Figure S4

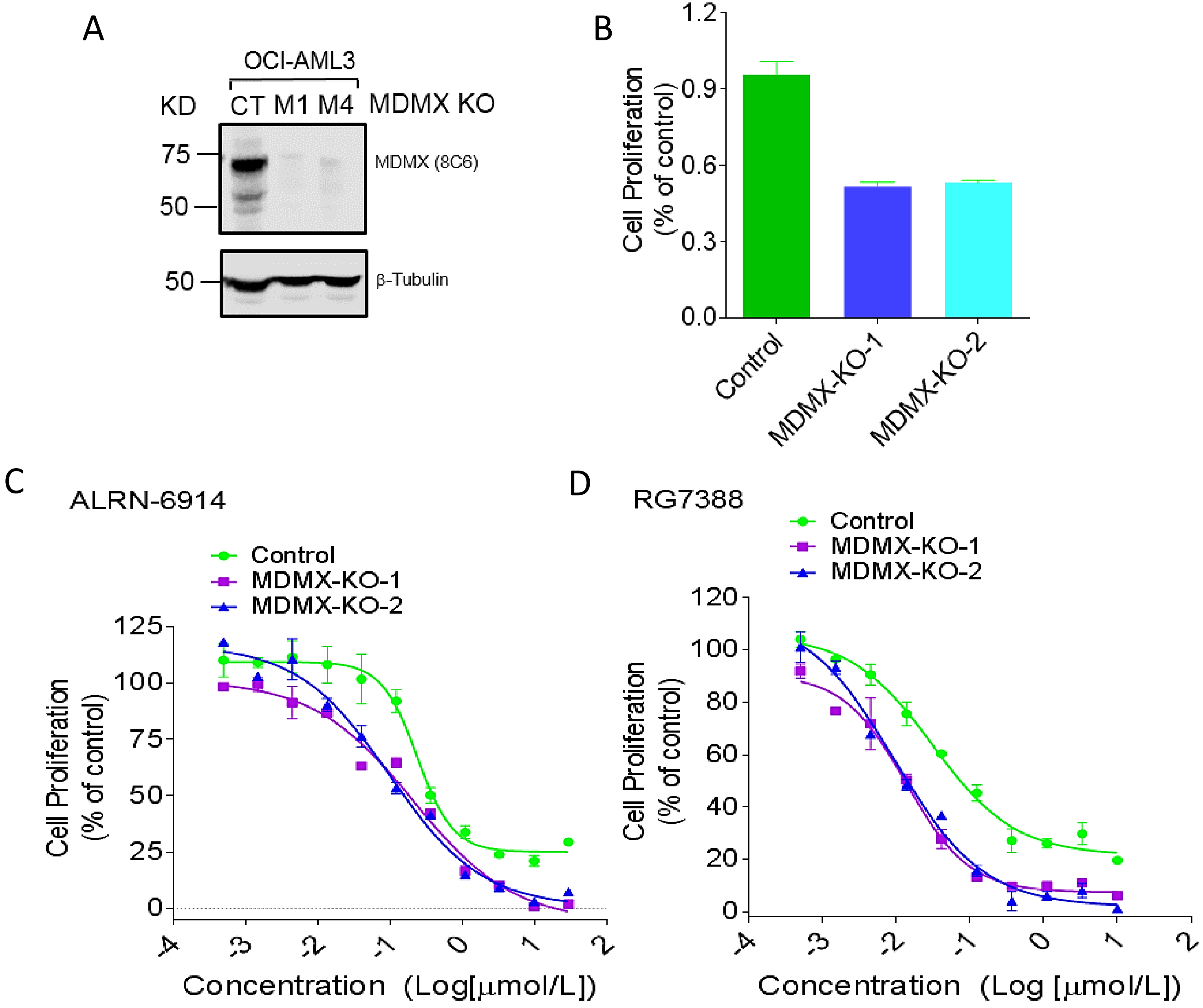

A
OCI-AML3
KD
CT M1 M4 MDMX KO
75
50
MDMX (8C6)
50
β-Tubulin
B
1.2
0.9
0.6
0.3
0.0
Cell Proliferation
(% of control)
Control
MDMX-KO-1
MDMX-KO-2
C
ALRN-6914
Control
MDMX-KO-1
MDMX-KO-2
125
100
75
50
25
0
Cell Proliferation
(% of control)
-4
-3
-2
-1
0
1
2
Concentration (Log[μmol/L])
D
RG7388
Control
MDMX-KO-1
MDMX-KO-2
120
100
80
60
40
20
0
Cell Proliferation
(% of control)
-4
-3
-2
-1
0
1
2
Concentration (Log [μmol/L])

Supplementary Table 1

Cell Line	Cancer Type	*TP53* Status	Source	HPV or SV40 infected	ALRN-6924 EC_{50} (μM)	RG7112 EC_{50} (μM)	RG7338 EC_{50} (μM)
**DU-4475**	Breast cancer	WT	Horizons		0.305	0.471	0.0711
**MEL-JUSO**	Melanoma	WT	Horizons		0.279	0.539	0.0424
**MEL-HO**	Melanoma	WT	Horizons		0.552	0.983	0.0546
**GRANTA-519**	Lymphoma	WT	Horizons		0.409	0.35	0.0371
**MDA-MB134-VI**	Breast cancer	WT	Horizons		2.29	4.51	0.123
**NH-6**	Neuroblastoma	WT	Horizons		0.299	0.551	0.0601
**SK-MEL-31**	Melanoma	WT	Horizons		1.94	1.03	0.133
**SK-N-SH**	Neuroblastoma	WT	Horizons		0.084	0.154	0.0071
**UACC-62**	Melanoma	WT	Horizons		1.01	0.516	0.00754
**OAW-42**	Ovarian cancer	WT	Horizons		0.546	0.567	0.164
**RMG-I**	Ovarian cancer	WT	Horizons		0.308	0.573	0.0034
**HCT-116**	Colon cancer	WT	Horizons		0.103	0.14	0.0202
**HEC-151**	endometrial cancer	WT	Horizons		0.239	1.64	0.522
**HEC-265**	endometrial cancer	WT	Horizons		0.544	1.6	0.243
**HUH-6 Clone 5**	Liver cancer	WT	Horizons		0.194	0.293	0.0188
**IST-MES1**	Lung cancer	WT	Horizons		0.814	0.974	>30
**KP-N-RT-BM-1**	Neuroblastoma	WT	Horizons		0.202	0.118	0.0224
**KP-N-S19s**	Neuroblastoma	WT	Horizons		0.031	0.22	0.00921
**LoVo**	Colon cancer	WT	Horizons		0.258	0.516	0.0245
**LS-174T**	Colon cancer	WT	Horizons		0.135	0.656	0.0157
**MSTO-211H**	Lung cancer	WT	Horizons		0.048	0.0753	0.00496
**MV-4-11**	AML	WT	Horizons		0.169	0.123	0.0226
**NCI-H929**	multiple myeloma	WT	Horizons		0.618	0.383	0.0351
**PA-1**	Ovarian cancer	WT	Horizons		0.135	0.526	0.0682
**WM-115**	Melanoma	WT	Horizons		0.369	0.661	0.108
**COLO-205**	Colon cancer	WT	Horizons		0.275	0.362	0.0142
**COLO-849**	Melanoma	WT	Horizons		0.619	1.21	0.0364
**NCI-H28**	Lung cancer	WT	Horizons		2.22	0.885	>30
**Colo-678**	Colon cancer	WT	Horizons		0.179	1.44	3.15
**SK-MEL-5**	Melanoma	WT	Horizons		0.585	1.88	>30
**COLO-792**	Melanoma	WT	Horizons		0.605	1.76	0.0372
**SK-MEL-24**	Melanoma	WT	Horizons		2.37	2.31	0.0165
**KP-4**	Pancreatic cancer	WT	Horizons		>30	13.2	>30
**C3A**	Liver cancer	WT	Horizons		0.069	0.389	0.00449
**A101D**	Melanoma	WT	Horizons		1.3	1.54	>30
**C32**	Melanoma	WT	Horizons		0.683	>30	>30
**HCC1395**	Breast cancer	Mutant	Horizons		11	11.4	11.9
**MDA-MB-361**	Breast cancer	Mutant	Horizons		>30	12.6	12.8
**HCC2218**	Breast cancer	Mutant	Horizons		>30	8.945334	30
**HCC1937**	Breast cancer	Mutant	Horizons		>30	7.57	7.09
**IGR-37**	Melanoma	Mutant	Horizons		>30	>30	>30
**Au565**	Breast cancer	Mutant	Horizons		>30	4.61	3.09
**BT-474**	Breast cancer	Mutant	Horizons		>30	11.2	7.15

**OCI-Ly7**	Lymphoma	Mutant	Horizons	>30	10.1	>30
**SK-BR-3**	Breast cancer	Mutant	Horizons	>30	11.1	6.65
**SK-MEL-2**	Melanoma	Mutant	Horizons	>30	>30	>30
**SK-MEL-30**	Melanoma	Mutant	Horizons	>30	13.4	>30
**SNU-387**	Liver cancer	Mutant	Horizons	19.3	>30	>30
**SNU-398**	Liver cancer	Mutant	Horizons	10.9	3.44	3.13
**SNU-423**	Liver cancer	Mutant	Horizons	15.7	6.94	30
**SNU-449**	Liver cancer	Mutant	Horizons	>30	>30	>30
**ECC12**	Gastric cancer	Mutant	Horizons	>30	11.4	>30
**EVSA-T**	Breast cancer	Mutant	Horizons	9.49	6.46	>30
**Hs 766T**	Pancreatic cancer	Mutant	Horizons	>30	12.9	>30
**MDA-MB-436**	Breast cancer	Mutant	Horizons	14.6	>30	>30
**OCI-Ly1**	Lymphoma	Mutant	Horizons	30	10.5	>30
**OE33**	esophageal cancer	Mutant	Horizons	16.7	4.27	>30
**Panc 02.03**	Pancreatic cancer	Mutant	Horizons	>30	7.34	>30
**Panc 04.03**	Pancreatic cancer	Mutant	Horizons	>30	8.2	>30
**PANC-1**	Pancreatic cancer	Mutant	Horizons	>30	9.21	15.4
**IA-LM**	NSC lung cancer	Mutant	Horizons	>30	12.2	15.5
**Karpas-422**	Lymphoma	Mutant	Horizons	>30	12.5	>30
**Mino**	Lymphoma	Mutant	Horizons	12.4	6.03	>30
**REC-1**	Lymphoma	Mutant	Horizons	20.2	2.65	>30
**A2058**	Melanoma	Mutant	Horizons	>30	16.1	>30
**AsPC-1**	Pancreatic cancer	Mutant	Horizons	>30	9.74	11.9
**Caov-3**	Ovarian cancer	Mutant	Horizons	>30	8.35	>30
**Caov-4**	Ovarian cancer	Mutant	Horizons	>30	14.7	>30
**COLO-679**	Melanoma	Mutant	Horizons	>30	7.65	8.97
**COLO-684**	endometrial cancer	Mutant	Horizons	9.71	12.6	>30
**COLO-741**	Melanoma	Mutant	Horizons	>30	12.2	>30
**EBC-1**	NSC lung cancer	Mutant	Horizons	>30	6.39	>30
**ECC10**	Gastric cancer	Mutant	Horizons	>30	7.48	14.6
**KG-1**	erythroleukemia	Mutant	Horizons	>30	16.2	>30
**KM12**	Colon cancer	Mutant	Horizons	>30	6.73	10.8
**LS-123**	Colon cancer	Mutant	Horizons	>30	>30	>30
**Mia PaCa-2**	Pancreatic cancer	Mutant	Horizons	>30	>30	>30
**NCI-H508**	Colon cancer	Mutant	Horizons	>30	6.6	11.4
**OVCAR-3**	Ovarian cancer	Mutant	Horizons	>30	13.4	>30
**RPMI-7951**	Melanoma	Mutant	Horizons	>30	12.2	>30
**SF126**	Brain cancer	Mutant	Horizons	>30	13.9	>30
**SK-OV-3**	Ovarian cancer	Mutant	Horizons	>30	15	>30
**SW480**	Colon cancer	Mutant	Horizons	>30	11.2	>30
**MPP-89**	Lung cancer	Mutant	Horizons	>30	>30	>30
**NCI-H2452**	Lung cancer	Mutant	Horizons	>30	13.7	>30
**HEC-6**	endometrial cancer	Mutant	Horizons	>30	10.7	13.2
**PANC-08-13**	Pancreatic cancer	Mutant	Horizons	>30	3.47	>30
**NCI-H838**	NSC lung cancer	Mutant	Horizons	>30	>30	>30
**HEC-108**	endometrial cancer	WT	Horizons	0.291	2.94	>30
**Daudi**	Lymphoma	Mutant	Eurofins	0.1		
**SW1353**	Bone	Mutant	Eurofins	0.179		

**MOLT-16**	Leukemia	Mutant	Eurofins	0.35
**U266B1**	Myeloma	Mutant	Eurofins	8.8
**SW-13**	Adrenal gland	Mutant	Eurofins	9.13
**SK-NEP-1**	Kidney	Mutant	Eurofins	9.47
**AN3 CA**	Endometrium	Mutant	Eurofins	10.1
**HLE**	Liver	Mutant	Eurofins	10.5
**DMS114**	Lung	Mutant	Eurofins	10.6
**Cal 27**	Mouth	Mutant	Eurofins	11
**MHH-PREB-1**	Lymphoma	Mutant	Eurofins	11
**HMCB**	Skin	Mutant	Eurofins	11.7
**HS 746T**	Stomach	Mutant	Eurofins	11.7
**DMS273**	Lung	Mutant	Eurofins	11.8
**RL95-2**	Uterus	Mutant	Eurofins	11.9
**RD**	Soft tissue	Mutant	Eurofins	12.1
**RKOE6**	Colon	Mutant	Eurofins	12.1
**SJRH30**	Sarcoma	Mutant	Eurofins	12.2
**CHL-1**	Skin	Mutant	Eurofins	12.5
**SK-N-AS**	CNS	Mutant	Eurofins	12.5
**Detroit 562**	Pharynx	Mutant	Eurofins	12.8
**SW1783**	Brain	Mutant	Eurofins	21.1
**Hs 578T**	Breast	Mutant	Eurofins	23.9
**5637**	Bladder	Mutant	Eurofins	>30
**639-V**	Ureter	Mutant	Eurofins	>30
**647-V**	Bladder	Mutant	Eurofins	>30
**786-O**	Kidney	Mutant	Eurofins	>30
**A431**	Vulva	Mutant	Eurofins	>30
**A-673**	Bone	Mutant	Eurofins	>30
**ARH-77**	Myeloma	Mutant	Eurofins	>30
**BE(2)C**	CNS	Mutant	Eurofins	>30
**BFTC-905**	Bladder	Mutant	Eurofins	>30
**BHT-101**	Thyroid	Mutant	Eurofins	>30
**BM-1604**	Prostrate	Mutant	Eurofins	>30
**BT20**	Breast	Mutant	Eurofins	>30
**BT-549**	Breast	Mutant	Eurofins	>30
**BxPC-3**	Pancreas	Mutant	Eurofins	>30
**C-33A**	Cervix	Mutant	Eurofins	>30
**CAL-62**	Thyroid	Mutant	Eurofins	>30
**Calu1**	Lung	Mutant	Eurofins	>30
**Calu6**	Lung	Mutant	Eurofins	>30
**CAMA-1**	Breast	Mutant	Eurofins	>30
**Capan-1**	Pancreas	Mutant	Eurofins	>30
**Capan-2**	Pancreas	Mutant	Eurofins	>30
**CCRFCEM**	Leukemia	Mutant	Eurofins	>30
**CEM-C1**	Leukemia	Mutant	Eurofins	>30
**CFPAC-1**	Pancreas	Mutant	Eurofins	>30
**CGTH-W-1**	Thyroid	Mutant	Eurofins	>30
**ChaGoK1**	Lung	Mutant	Eurofins	>30
**Colo 201**	Colon	Mutant	Eurofins	>30

**Colo 205**	Colon	Mutant	Eurofins	>30
**Colo 320 HSR**	Colon	Mutant	Eurofins	>30
**Colo 320DM**	Colon	Mutant	Eurofins	>30
**COR-L23**	Lung	Mutant	Eurofins	>30
**Daoy**	CNS	Mutant	Eurofins	>30
**DB**	Lymphoma	Mutant	Eurofins	>30
**DLD-1**	Colon	Mutant	Eurofins	>30
**DMS53**	Lung	Mutant	Eurofins	>30
**DU145**	Prostrate	Mutant	Eurofins	>30
**EB-3**	Lymphoma	Mutant	Eurofins	>30
**EFM-19**	Breast	Mutant	Eurofins	>30
**EM-2**	Leukemia	Mutant	Eurofins	>30
**ES-2**	Ovary	Mutant	Eurofins	>30
**FaDu**	Pharynx	Mutant	Eurofins	>30
**HCT-15**	Colon	Mutant	Eurofins	>30
**HEC-1-A**	Endometrium	Mutant	Eurofins	>30
**HEL-92-1-7**	Leukemia	Mutant	Eurofins	>30
**HLF**	Liver	Mutant	Eurofins	>30
**HOS**	Bone	Mutant	Eurofins	>30
**HPAF-II**	Pancreas	Mutant	Eurofins	>30
**HT**	Lymphoma	Mutant	Eurofins	>30
**HT1376**	Bladder	Mutant	Eurofins	>30
**HT-29**	Colon	Mutant	Eurofins	>30
**HT-3**	Cervix	Mutant	Eurofins	>30
**HuCCT1**	Biliary duct	Mutant	Eurofins	>30
**HuP-T4**	Pancreas	Mutant	Eurofins	>30
**J82**	Bladder	Mutant	Eurofins	>30
**J-RT3-T3-5**	Leukemia	Mutant	Eurofins	>30
**Jurkat**	Leukemia	Mutant	Eurofins	>30
**K562**	Leukemia	Mutant	Eurofins	>30
**KATO III**	Stomach	Mutant	Eurofins	>30
**KHOS-240S**	Bone	Mutant	Eurofins	>30
**KLE**	Uterus	Mutant	Eurofins	>30
**L-428**	Lymphoma	Mutant	Eurofins	>30
**LS1034**	Large intestine	Mutant	Eurofins	>30
**MC-IXC**	CNS	Mutant	Eurofins	>30
**MDA MB 231**	Breast	Mutant	Eurofins	>30
**MDA MB 453**	Breast	Mutant	Eurofins	>30
**MDA MB 468**	Breast	Mutant	Eurofins	>30
**MEG01**	Leukemia	Mutant	Eurofins	>30
**MeWo**	Skin	Mutant	Eurofins	>30
**MG-63**	Bone	Mutant	Eurofins	>30
**NCI-H295R**	Adrenal gland	Mutant	Eurofins	>30
**NCIH441**	Lung	Mutant	Eurofins	>30
**NCIH446**	Lung	Mutant	Eurofins	>30
**NCI-H520**	Lung	Mutant	Eurofins	>30
**NCI-H596**	Lung	Mutant	Eurofins	>30
**NCI-H661**	Lung	Mutant	Eurofins	>30

**NCI-H69**	Lung	Mutant	Eurofins	>30
**NCI-H747**	caecum	Mutant	Eurofins	>30
**OE19**	Esophagus	Mutant	Eurofins	>30
**OE21**	oesophagus	Mutant	Eurofins	>30
**PC-3**	Prostrate	Mutant	Eurofins	>30
**Raji**	Lymphoma	Mutant	Eurofins	>30
**Ramos (RA 1)**	Lymphoma	Mutant	Eurofins	>30
**RPMI 8226**	Myeloma	Mutant	Eurofins	>30
**SaOS2**	Bone	Mutant	Eurofins	>30
**SCaBER**	Urinary Tract	Mutant	Eurofins	>30
**SCC-25**	Mouth	Mutant	Eurofins	>30
**SCC-4**	mouth	Mutant	Eurofins	>30
**SCC-9**	mouth	Mutant	Eurofins	>30
**SHP-77**	Lung	Mutant	Eurofins	>30
**SK-LMS-1**	Soft tissue	Mutant	Eurofins	>30
**SK-MEL-1**	Skin	Mutant	Eurofins	>30
**SK-MEL-28**	Skin	Mutant	Eurofins	>30
**SK-MEL-3**	Skin	Mutant	Eurofins	>30
**SKMES1**	Lung	Mutant	Eurofins	>30
**SK-N-DZ**	CNS	Mutant	Eurofins	>30
**SK-N-FI**	CNS	Mutant	Eurofins	>30
**SKOV3**	Ovary	Mutant	Eurofins	>30
**SK-UT-1**	Soft tissue	Mutant	Eurofins	>30
**SNB-19**	CNS	Mutant	Eurofins	>30
**SNU-16**	Stomach	Mutant	Eurofins	>30
**SNU-5**	Stomach	Mutant	Eurofins	>30
**ST486**	Lymphoma	Mutant	Eurofins	>30
**SU.86.86**	Pancreas	Mutant	Eurofins	>30
**SW1088**	CNS	Mutant	Eurofins	>30
**SW1417**	Colon	Mutant	Eurofins	>30
**SW1463**	Rectum	Mutant	Eurofins	>30
**SW403**	Colon	Mutant	Eurofins	>30
**SW579**	Thyroid	Mutant	Eurofins	>30
**SW620**	Colon	Mutant	Eurofins	>30
**SW684**	Soft tissue	Mutant	Eurofins	>30
**SW837**	Rectum	Mutant	Eurofins	>30
**SW872**	Soft tissue	Mutant	Eurofins	>30
**SW900**	Lung	Mutant	Eurofins	>30
**SW948**	Colon	Mutant	Eurofins	>30
**SW954**	Vulva	Mutant	Eurofins	>30
**SW962**	Vulva	Mutant	Eurofins	>30
**T24**	Urinary Tract	Mutant	Eurofins	>30
**T47D**	Breast	Mutant	Eurofins	>30
**T98G**	CNS	Mutant	Eurofins	>30
**TCCSUP**	Urinary Tract	Mutant	Eurofins	>30
**Thp1**	Leukemia	Mutant	Eurofins	>30
**U-138MG**	Brain	Mutant	Eurofins	>30
**UM-UC-3**	Bladder	Mutant	Eurofins	>30

**WiDr**	Colon	Mutant	Eurofins	>30
**YAPC**	Pancreas	Mutant	Eurofins	>30
**AGS**	Stomach	WT	Eurofins	0.03
**D283 Med**	CNS	WT	Eurofins	0.04
**U-87 MG**	CNS	WT	Eurofins	0.04
**DBTRG-05MG**	CNS	WT	Eurofins	0.05
**SNU-1**	Stomach	WT	Eurofins	0.05
**G-402**	Kidney	WT	Eurofins	0.05
**G-401**	Kidney	WT	Eurofins	0.06
**DOHH-2**	Lymphoma	WT	Eurofins	0.06
**A172**	CNS	WT	Eurofins	0.07
**A375**	Skin	WT	Eurofins	0.07
**H4**	CNS	WT	Eurofins	0.08
**SW982**	Sarcoma	WT	Eurofins	0.0783
**HCT-8**	Colon	WT	Eurofins	0.09
**CRO-AP2**	Lymphoma	WT	Eurofins	0.09
**Caki-1**	Kidney	WT	Eurofins	0.1
**KPL-1**	Breast	WT	Eurofins	0.106
**CHP-212**	CNS	WT	Eurofins	0.12
**SR**	Lymphoma	WT	Eurofins	0.12
**Hs 294T**	Skin	WT	Eurofins	0.125
**U2OS**	Bone	WT	Eurofins	0.13
**HT-1080**	Soft tissue	WT	Eurofins	0.13
**NCI-H292**	Lung	WT	Eurofins	0.13
**BV-173**	Leukemia	WT	Eurofins	0.14
**RKO-AS45-1**	Colon	WT	Eurofins	0.15
**MALME3M**	Skin	WT	Eurofins	0.16
**A204**	Soft tissue	WT	Eurofins	0.19
**A498**	Kidney	WT	Eurofins	0.2
**RKO**	Colon	WT	Eurofins	0.21
**JEG-3**	Placenta	WT	Eurofins	0.21
**MOLT-3**	Leukemia	WT	Eurofins	0.21
**COR-L105**	Lung	WT	Eurofins	0.21
**COLO 829**	Skin	WT	Eurofins	0.22
**C32TG**	Skin	WT	Eurofins	0.23
**Caki-2**	Kidney	WT	Eurofins	0.25
**SH-4**	Skin	WT	Eurofins	0.26
**22Rv1**	Prostrate	WT	Eurofins	0.27
**HepG2**	Liver	WT	Eurofins	0.27
**NALM-6**	Leukemia	WT	Eurofins	0.28
**MES-SA**	Soft tissue	WT	Eurofins	0.31
**MT-3**	Breast	WT	Eurofins	0.31
**SJSA1**	Bone	WT	Eurofins	0.33
**DK-MG**	CNS	WT	Eurofins	0.33
**A549**	Lung	WT	Eurofins	0.38
**CML-T1**	Leukemia	WT	Eurofins	0.39
**MCF7**	Breast	WT	Eurofins	0.41
**HT-1197**	Bladder	WT	Eurofins	0.411

**Hs 695T**	Skin	WT	Eurofins		0.44
**RPMI 6666**	Lymphoma	WT	Eurofins		0.45
**ACHN**	Kidney	WT	Eurofins		0.47
**A427**	Lung	WT	Eurofins		0.55
**BC-1**	Lymphoma	WT	Eurofins		0.55
**SW48**	Colon	WT	Eurofins		0.552
**JAR**	Placenta	WT	Eurofins		0.66
**LNCaP**	Prostrate	WT	Eurofins		0.77
**769-P**	Kidney	WT	Eurofins		0.77
**NCI-H460**	Lung	WT	Eurofins		1.09
**BeWo**	Placenta	WT	Eurofins		1.66
**Y79**	Eye	WT	Eurofins		2.15
**CCF-STTG1**	CNS	WT	Eurofins		3.6
**BPH1**	Prostrate	WT	Eurofins	Yes	>30
**C-4 I**	Cervix	WT	Eurofins	Yes	>30
**C-4 II**	Cervix	WT	Eurofins	Yes	>30
**DoTc2 4510**	Cervix	WT	Eurofins	Yes	>30
**HeLa**	Cervix	WT	Eurofins	Yes	>30
**SiHa**	Cervix	WT	Eurofins	Yes	>30

Supplementary Table 2.

MCF-7 ALRN-6924 + Paclitaxel vs Control IPA analysis – Top Canonical Pathways

Name	p-value
p53 Signaling	9.59E-06
Estrogen-mediated S-phase Entry	5.82E-05
Cell-cycle: G1/S Checkpoint Regulation	3.92E-04
Small Cell Lung Cancer Signaling	4.40E-04
Cyclins and Cell cycle Regulation	5.73E-04
